# Prevalence of Meniscal and Articular Surface Injuries in Patients Undergoing Primary and Revision Anterior Cruciate Ligament Reconstruction: A Retrospective Analysis

**DOI:** 10.7759/cureus.85106

**Published:** 2025-05-30

**Authors:** Juanjose Valderrama, Agustín León, Rodrigo Hernández, Marcelo Acevedo, Xabier Carredano, Eduardo Gardella, Adolfo Mena, Cristóbal Vigueras, Gunther Redenz

**Affiliations:** 1 Orthopedics, Clinica Indisa, Santiago, CHL; 2 Orthopedics, Mutual de Seguridad Asociación Chilena de Seguridad, Santiago, CHL; 3 Research, Clinica Indisa, Santiago, CHL; 4 Faculty of Medicine, Universidad Andres Bello, Santiago, CHL

**Keywords:** anterior cruciate ligament (acl), anterior cruciate ligament reconstruction (aclr), articular cartilage, meniscal tear, orthopedics sports, revision of surgery

## Abstract

Background

Patients undergoing anterior cruciate ligament reconstruction frequently present with concomitant meniscal or articular cartilage injuries. In revision anterior cruciate ligament reconstruction (ACLR), the prevalence of articular cartilage damage is higher compared to primary reconstruction. However, limited data are available regarding the extent and distribution of cartilage damage by articular surface.

Objective

To determine the differences in the prevalence and characteristics of meniscal tears and articular surface injuries between patients who underwent primary ACLR or ACLR revision.

Methods

A cross-sectional study of patients undergoing ACL surgery between 2017 and 2023. Patient characteristics and arthroscopic findings on the location and type of meniscal injury, as well as the degree of chondral lesion, were recorded by the surgeon. A chi-square test was used to determine differences between reconstruction and revision patients, and a simple logit model was used to estimate the risk of lesions.

Results

Around 527 surgeries were analyzed: 92.79% reconstructions and 7.21% revisions. Meniscal tear prevalence was 69.83% and was more frequent in the lateral menisci. Complex and degenerative tears were more frequent in revision patients. Overall, articular surface chondral damage prevalence was 24.48% and was affected by the meniscal condition and revision status (OR=3.53 (95% CI: 1.72‒7.28)), up to 42.72% and 66.67% in reconstruction and revision patients, respectively.

Discussion

Lateral and medial meniscus injuries were common, with complex tears more frequent in revisions. Chondral lesions were more often detected when both menisci were torn or when the lateral meniscus alone was affected. The medial femoral condyle emerged as the most commonly injured articular surface. Although conflicting evidence exists regarding meniscal tear incidence in revision cases, degenerative and multidirectional tears appeared to be more prevalent. Future research is needed to clarify injury patterns, improve detection of minor chondral damage, and optimize surgical timing.

Conclusion

Meniscal tears (up to 69.83%) and articular cartilage injuries (24.48%) are common in patients undergoing primary or revision ACL reconstructions. Revision procedures, sex, age, and meniscal tears increase injury likelihood. The medial femoral condyle is most often affected, while the lateral tibial plateau is least involved

## Introduction

Approximately 80% of surgically treated knee ligament injuries involve anterior cruciate ligament (ACL) tears [1]. Considering patients’ age and physical activity, reconstruction surgery is preferred in the acute stage in at least 75% of individuals [2]. ACL deficiency can lead to the early onset of degenerative conditions, such as radiographic osteoarthritis (OA) [3].

It is common to discover previously undiagnosed joint injuries during reconstruction surgery. Up to 45% of ACL ruptures are associated with additional ligamentous, meniscal, or chondral lesions [4]. Chondral or meniscal lesions would increase the presence of radiological OA and worsen functional capacity compared to healthy knees [5,6], and they also predict worse clinical outcomes [7]. It has been reported that the odds ratio (OR) for any intra-articular lesion among individuals who delay ACL reconstruction (ACLR) by 12 months or more is 1.90 (95% CI 1.33-2.71) [8].

Abundant literature describes the frequency of meniscal damage, which varies depending on the sport, age, and sex [9-11]. Articular surface lesions may result from joint instability [12] and the long-term loss of meniscal integrity [13]. Approximately 80% of chondral injuries are associated with meniscal damage in the same compartment [14]. A recent meta-analysis [13] concluded that the distribution of chondral is balanced between the medial and lateral compartments of the knee. However, reports on the prevalence of tears in ACLR revision (ACLR-R) are mixed, and it remains unclear whether there is a higher prevalence [4,7].

It is known that differences in the prevalence of articular injuries exist between ACLR and ACLR-R [4]. A recent study found a higher prevalence of articular cartilage lesions at the time of ACLR-R in followed-up patients (15.5% vs. 7.0%) [15]. Although reports including the extent of the lesion by surface are infrequent [7]. The objectives of this study are to determine the differences in the prevalence and characteristics of meniscal tears and articular surface injuries between patients who underwent primary ACLR or ACLR-R; to estimate the association between the type of surgery (ACLR vs. ACLR-R) and the presence of articular lesions.

## Materials and methods

Study design

A cross-sectional prevalence study was conducted in accordance with the Strengthening the Reporting of Observational Studies in Epidemiology (STROBE) guidelines at Clinica Indisa, located in Santiago, Chile [16]. A database generated by knee surgeons from a sports medical center was analyzed retrospectively. This database included a consecutive registry of all the knee ligament surgeries performed between April 2017 and April 2023. The Ethical-Scientific Committee of the care center approved the protocol (authorization number 0022021).

The eligibility criteria were as follows: all patients whose surgical record indicated ACLR or ACLR-R were included in the analysis. Patients who underwent augmentation or partial reconstruction of the ACL were excluded.

Data collection

Protocols for ligament reconstruction surgery, the type of meniscal injury and its treatment, the type of chondral injury, sex, and age were recorded by the treating surgeon. As primary variables, evaluated via arthroscopic observation, the location and type of meniscal injury, as well as the degree of chondral injury according to the International Cartilage Repair Society (ICRS) [17], were documented as follows: Grade 0 signifies the absence of a lesion; Grade I represents superficial lesions, such as chondromalacia, superficial fissures, and soft indentations; Grade II includes lesions that extend to less than 50% of the cartilage depth; Grade III indicates cartilage defects that extend beyond 50% of the cartilage depth and may also involve the calcified layer, but not the subchondral bone; and Grade IV, lesions that extend through the subchondral bone.

Statistical analysis

The characteristics of the surgeries performed and the findings of meniscal and chondral injury during arthroscopy were reported as frequencies. The degree of chondral injury on the articular surfaces, based on the ICRS classification, was summarized as quartiles. The association between the percentage of meniscectomies and the meniscus affected (medial or lateral) was assessed by measuring the effect size using Cohen’s d after applying a nonparametric test to compare means. Differences in categorical data were determined by chi-square or Fisher’s exact test, following verification of statistical assumptions.

The relative risk ratio of meniscal tears was estimated using a multinomial (polytomous) logistic regression, adjusting for age and sex, with revision as the independent variable. The risk of presenting chondral lesions was estimated using logistic regression adjusted for age and sex, with the presence of meniscal injury (none, medial meniscus, lateral meniscus, or both) and revisions as the independent variables. These analyses were performed by a researcher independent of the surgical team. All analyses were performed using Stata 15® software (StataCorp LLC, College Station, TX), with a significance level (alpha) set at 5%.

## Results

A total of 527 surgeries were analyzed out of 545. Thirteen observations were excluded because they involved other ligamentous injuries, and five were excluded due to missing treatment or ligament data. Among the analyzed cases, 422 (80.23%) were men and 104 (19.77%) were women. The mean age was 32.37 years (SD: 9.64), ranging from 15 to 66 years. Of the total cases, 489 (92.79%) were primary reconstructions, while 38 (7.21%) were revision procedures.

Regarding graft selection, semitendinosus-gracilis tendon was used in 404 cases (76.66%), while bone-patellar-tendon-bone (BTB), quadricipital tendon, and allograft were used in 47 (8.92%), 57 (10.82%), and 19 (3.61%) cases, respectively. A detailed description of the ACLR and ACLR-R groups is presented in Table 1.

**Table 1:** Patient characteristics Quantitative variables are presented as mean (standard deviation), while qualitative variables are presented as n= (%); *: difference between reconstruction and revision ACL: anterior cruciate ligament; STG: semitendinosus-gracilis; BTB: bone-patellar-tendon-bone; Q: quadricipital

Characteristics	ACL Reconstruction		ACL Revision	
	Autograft (n=475)	Allograft (n=14)	Autograft (n=33)	Allograft (n=5)
Age	32.07 (9.38)	49.21 (6.86)	30.09 (7.70)	29.8 (10.16)
Male	380 (80.17%)	11 (78.57%)	27 (81.82%)	4 (80.00%)
Right side	260 (54.85%)	8 (57.14%)	19 (57.58%)	3 (60.00%)
STG graft* (p-value <0.001)	403 (82.41%)	—	1 (2.63%)	—
BTB graft* (p-value <0.001)	30 (6.13%)	—	17 (44.74%)	—
Q graft* (p-value <0.001)	42 (8.59%)	—	15 (39.47%)	—

The overall prevalence of meniscal injuries was 69.83% (368/527). Among these, 97 cases (18.41%) involved the medial meniscus, 168 (31.88%) affected the lateral meniscus, and 103 (19.54%) involved both menisci. A comprehensive description of ACLR and ACLR-R is presented in Table 2.

**Table 2:** Meniscal tear description in ACLR and ACLR-R patients Categories were not exclusionary, a double count exists with both menisci. WW: white white; WR: white red; RR: red red; PH: posterior horn; AH: anterior horn; ACLR-R: anterior cruciate ligament reconstruction-revision *: statistical difference between lateral and medial menisci; †: statistical difference between medial menisci of reconstruction and revision patients; ‡: statistical difference between the lateral menisci of reconstruction and revision patients

Description	Categories	Reconstruction (n=489)		Revision (n=38)	
		Lateral menisci (50.92%, n=249)	Medial menisci (38.85%, n=190)	Lateral menisci (57.89%, n=22)	Medial menisci (28.95%, n=11)
Injury zone* (p-value<0.01)	WW	80 (33.76%)	18 (9.42%)	4 (18.18%)	1 (9.09%)
	WR	143 (60.34%)	100 (52.36%)	15 (68.18%)	7 (63.64%)
	RR	14 (5.91%)	73 (38.22%)	3 (13.64%)	3 (27.27%)
Topography* (p-value<0.01)	PH	116 (46.59%)	81 (42.63%)	6 (27.27%)	6 (54.55%)
	Body-PH	60 (24.10%)	68 (35.79%)	10 (45.45%)	3 (27.27%)
	Body	50 (20.08%)	11 (5.79%)	3 (13.64%)	1 (9.09%)
	Body-AH	8 (3.21%)	1 (0.53%)	0	0
	AH	6 (2.41%)	3 (1.58%)	2 (9.09%)	0
	PH-Body-AH	9 (3.61%)	26 (13.68%)	1 (4.55%)	1 (9.09%)
Type of tear* (p-value<0.01) †(p<0.01) ‡(p<0.01)	Degenerative	20 (4.09%)	10 (2.04%)	3 (7.89%)	2 (5.26%)
	Bucket handle	5 (1.02%)	34 (6.95%)	4 (10.53%)	1 (2.63%)
	Multidirectional	20 (4.09%)	17 (3.48%)	2 (5.26%)	4 (10.53%)
	Radial	58 (11.86%)	8 (1.64%)	2 (5.26%)	0
	Longitudinal	93 (19.02%)	100 (20.44%)	6 (15.79%)	1 (2.63%)
	Flap/oblique	25 (5.11%)	7 (1.43%)	3 (7.89%)	1 (2.63%)
	Disinsertion	21 (4.29%)	3 (0.61%)	1 (2.63%)	0
	Ramp	0	9 (1.84%)	1 (2.63%)	1 (2.63%)
	Post-meniscectomy	1 (0.20%)	1 (0.20%)	0	1 (2.63%)
	Without tear	240 (49.08%)	300 (61.35%)	16 (42.11%)	27 (71.05%)
Resolution* (p-value<0.01)	Meniscectomy	145 (64.44%)	71 (42.01%)	14 (63.64%)	7 (63.64%)
	Suture	61 (27.11%)	94 (55.62%)	5 (22.73%)	4 (36.36%)
	Reinsertion	18 (8.00%)	3 (1.78%)	3 (13.64%)	0

Significant differences were observed in the injury zone site, topography, tear type, and resolution between the medial and lateral menisci (p<0.01, chi-square test). Notably, lateral meniscus tears tended to be more concentrically distributed, whereas posterior horn lesions were similarly prevalent in both menisci. Full-body tears were more common in the medial meniscus. Additionally, half of the lateral menisci exhibited signs of damage, compared to only one-third of the medial menisci. When comparing meniscal injuries between primary reconstruction and revision procedures, the only significant difference was the tear type.

The mean percentage of meniscectomy was 39.19% (SD: 26.23%, n=43) for the medial meniscus and 19.42% (SD: 14.61%, n=106) for the lateral meniscus. This difference was statistically significant (p<0.001), with a post-hoc power of 0.99 and an effect size calculated using Cohen's d of 1.06 (95% CI: 0.68-1.43).

Around 129 records (24.48%) presented one or more articular surface lesions in the sample. Sorted by meniscal injury, the prevalence of articular surface damage was 13.84% in cases without meniscal injury, 21.65% in cases with medial meniscus injury, 25.00% in those with lateral meniscus injury, and 42.72% in cases involving both menisci. The differences in the proportions of articular surface damage among these groups were statistically significant (p<0.001, chi-square test). Primary ACLR had a lower proportion of articular surface injuries compared to ACLR-R (p=0.003, chi-square test). In ACLR-R cases, the prevalence of articular surface lesions reached 66.67% in patients with injuries affecting both menisci, whereas the prevalence was similar between those with lateral menisci and those without meniscal tears. The degree of chondral damage in different articular surfaces for both reconstruction and revision procedures is summarized in Table 3. Overall, 20.30% of cases presented concomitant meniscal tears and concomitant articular surface injuries.

**Table 3:** Articular surface injuries among ACLR and ACLR-R Data are reported as n (%) of non-missing values or as median (lower quartile, upper quartile) for continuous variables. ICRS: International Cartilage Repair Society; Q1: first quartile; Q3: third quartile; ACLR-R: anterior cruciate ligament reconstruction-revision *: statistical difference between meniscal injuries on ACLR patients; **: statistical difference between meniscal injuries on ACLR-R patients

Data	Reconstruction (n=489)								Revision (n=38)							
Articular surface	Medial menisci (n=92)		Lateral menisci (n=152)		Both (n=97)		Without (n=148)		Medial menisci (n=5)		Lateral menisci (n=16)		Both (n=6)		Without (n=11)	
	n= (%)	ICRS (Q1‒Q3)	n= (%)	ICRS (Q1‒Q3)	n= (%)	ICRS (Q1‒Q3)	n= (%)	ICRS (Q1‒Q3)	n= (%)	ICRS (Q1‒Q3)	n= (%)	ICRS (Q1‒Q3)	n= (%)	ICRS (Q1‒Q3)	n= (%)	ICRS (Q1‒Q3)
Lateral femoral condyle *(p<0.01)** (p<0.01)	2 (2.17%)	1.5 (1‒2)	12 (7.89%)	3 (2‒4)	12 (12.37%)	2 (1‒3)	3 (2.03%)	3 (3‒4)	1 (20.00%)	1 (1‒1)	3 (18.75%)	3 (1‒3)	1 (1.67%)	2 (2‒2)	1 (9.09%)	2 (2‒2)
Medial femoral condyle *(p<0.01) **(p<0.01))	12 (13.04%)	3 (3‒3)	14 (9.21%)	3 (2‒3)	24 (24.74%)	3 (2‒3)	9 (6.08%)	3 (2‒3)	3 (60.00%)	3 (3‒3)	3 (18.75%)	2 (1‒3)	3 (50.00%)	3 (2‒3)	2 (18.18%)	3 (2‒4)
Lateral tibial plateau *(p<0.01) **(p<0.01)	4 (4.35%)	1 (1‒1)	13 (8.55%)	2 (1‒3)	14 (14.43%)	2 (1‒2)	1 (0.68%)	2 (2‒2)	1 (20.00%)	1 (1‒1)	4 (25.00%)	2.5 (2‒3)	1 (16.67%)	2 (2‒2)	2 (18.18%)	2 (2‒2)
Medial tibial plateau *(p<0.01) **(p<0.01)	5 (5.43%)	2 (1‒2)	3 (1.97%)	2 (2‒3)	13 (13.40%)	2 (1‒2)	1 (0.68%)	2 (2‒2)	0 (0.00%)	‒	2 (12.50%)	1.5 (1‒2)	1 (16.67%)	3 (3‒3)	2 (18.18%)	2 (2‒2)
Patella *(p<0.01) **(p<0.01)	2 (2.17%)	3 (3‒3)	10 (6.58%)	2 (2‒3)	14 (14.43%)	3 (2‒3)	6 (4.05%)	3 (2‒3)	1 (20.00%)	3 (3‒3)	0 (0.00%)	‒	1 (16.67%)	2 (2‒2)	0 (0.00%)	‒
Trochlea	2 (2.17%)	3 (3‒3)	3 (1.97%)	3 (2‒3)	6 (6.19%)	3 (3‒4)	2 (1.35%)	3 (2‒4)	0 (0.00%)	‒	1 (6.25%)	3 (3‒3)	0 (0.00%)	‒	0 (0.00%)	‒

The multinomial logistic regression model for meniscal tears indicated a significant association with male sex and age in specific categories. The full model output is provided in Table 4. Furthermore, the presence of chondral lesions, adjusted for age and sex, was associated with an OR of 5.62 (95% CI: 2.97-10.67) in cases involving both menisci, 2.48 (95% CI: 1.35-4.55) in cases with lateral meniscus injury, and 1.60 (95% CI: 0.80-3.22) in cases with medial meniscus injury. Additionally, individuals undergoing revision procedures had an OR of 3.53 (95% CI: 1.72-7.28) compared to those undergoing primary ACL reconstruction.

**Table 4:** Multinomial logistic regression on meniscal tear The base outcome is defined as a female, zero years old, without a meniscal tear and primary reconstruction (anterior cruciate ligament reconstruction). Likelihood of the model 31.33, chi-square <0.01. RRR: relative risk ratio; cons: constant of the category

Meniscal tear	Predictor	RRR	95% conf. interval	p-value
Both injured	Age	1.01	0.98 ‒ 1.04	0.41
	Male	0.35	0.18 ‒ 0.68	<0.01
	Revision	0.85	0.30 ‒ 2.41	0.76
	Cons	0.56	0.23 ‒ 1.40	0.22
Lateral meniscus	Age	0.99	0.97 ‒ 1.01	0.37
	Male	0.36	0.20 ‒ 0.63	<0.01
	Revision	1.38	0.61 ‒ 3.12	0.44
	Cons	1.78	0.81 ‒ 3.93	0.15
Medial meniscus	Age	1.03	1.01 ‒ 1.06	0.01
	Male	0.65	0.35 ‒ 1.17	0.15
	Revision	0.79	0.26 ‒ 2.38	0.68
	Cons	0.23	0.09 ‒ 0.59	<0.01

## Discussion

This study aimed to describe the prevalence and assess the association of lesions at the time of primary (ACLR) or subsequent revision (ACLR-R) of ACLR. Lateral and medial meniscus injuries were observed in 50.92% and 38.85% of primary reconstructions, respectively, and in 57.89% and 28.95% of revisions. Articular surface lesions were more frequently associated with meniscal tears (42.72%) compared to isolated medial or lateral meniscal tears.

The prevalence of meniscal injuries concomitant to ACL deficit has been widely reported in the literature, ranging from 24.2% to 69% [2,4,14,18-22]. Time to surgery is a factor that may increase this prevalence, reaching up to 67%‒72.8% [14,18,21,23-25]. According to our statistical model, ACLR-R did not show a significant association with the presence of meniscal tears. This finding contrasts with previous statistical models; however, some studies have also concluded that meniscal injuries occur independently of revision status [7,26]. However, a well-established cohort study reported an increased prevalence of meniscal tears in revision cases [4]. The differences in injury location, tear morphology, and type of meniscal damage between ACLR and ACLR-R described in the literature are difficult to weigh due to the multifactorial nature of the ACL failure and variations in the surgical technique.

Regarding these injuries, the most frequent tear patterns in the medial meniscus were longitudinal (47%) and bucket handle (25%), similar to the findings reported by Mansori et al. (2018) [24]. In the lateral meniscus, longitudinal (33.8%) and radial tears (22.5%) were the most common, whereas bucket-handle tears had a low prevalence (1.6%). However, the literature describes a wide range of tear pattern distributions [18,23-25], highlighting the need for further research to assess the influence of various factors on each pattern.

In this series, the tear patterns differed in revision patients, with degenerative and complex tears, such as multidirectional tears, being more prevalent. It has been recognized that complex tears are associated with an increased risk of total or subtotal meniscectomy [27]. In our study, the degree of damage, assessed based on the percentage of meniscectomy, was higher in the lateral meniscus, which could be attributed to the distribution of tear patterns.

The location of injuries also varied between the two menisci. In the medial meniscus, the posterior horn was slightly more affected than the body, whereas in the lateral meniscus, two-thirds of the injuries were located in the body-contrary to previous reports [21,24]. Although these findings resemble those reported for the chronic group described by Hagino et al. [21], we attribute the difference not to the interval between injury and surgery but to the higher mean age of the group.

Concomitant articular surface injury reports observed by arthroscopy are also diverse. These injuries are detected in approximately 10% of patients when surgery is performed early [4,25]. However, when reconstruction is delayed, the detection rate increases to 20%-55% [14,25]. Similar to previous studies [4,8,14,28,29], the medial femoral condyle was the most commonly affected joint surface. There was a limited number of patients with chondral lesions of the lateral tibial plateau. Expanding the sample size in future studies would be valuable to investigate a potential association between these lesions and ipsilateral meniscal injuries.

Considering the overall prevalence, in ACLR cases, the lateral and medial compartment injuries were similar in patients with medial meniscal tears (6.53% and 18.48% in the lateral and medial compartments, respectively) and in those without tears (8.33% and 20.83%, respectively). However, in cases of lateral meniscal tears (48.08% and 32.69%, respectively) or combined medial and lateral meniscal tears (26.80% and 38.14%, respectively), the prevalence was higher. This pattern may not apply to ACLR-R, where the prevalence of articular surface injuries is higher than ACLR in all the surfaces. Notably, in several categories, the extent of articular surface injuries was greater in patients without meniscal tears. As observed in other studies [30], this finding may partly result from the underreporting of these injuries, particularly those classified as ICRS grades 1 and 2, which are typically considered minor. Future research should aim to improve the detection of minor articular cartilage injuries.

According to our statistical model, when both menisci were injured, the chances of articular surface lesions increased in the presence of bilateral meniscal tears or isolated lateral meniscal tears. The chances are 3.53 (95% CI: 1.72‒7.28) higher in the ACLR-R group. Differences in lesion prevalence between ACLR and ACLR-R prevalence are well documented [4,15]. However, to the best of our knowledge, studies reporting the severity of articular surface injuries in patients undergoing ACLR-R remain scarce [7]. The joint surfaces with the greatest degree of lesion were the trochlea and patella, while the medial or lateral tibial plateau and lateral femoral condyle exhibited the least severe lesions. Finally, 16.5% of the cases presented concomitant meniscal and chondral injuries, which aligns with findings previously reported for the general Norwegian population [31].

One limitation of this study is the absence of clinically relevant information, such as the cause of injury or the level of physical activity, due to its retrospective and descriptive design. Inclusion of such information would enhance the assessment of risk factors for severe concurrent injuries. Another limitation arises from the involvement of multiple specialists in the diagnostic process, potentially introducing variability in diagnostic criteria. An important strength, however, was the utilization of validated tools for both diagnosis and data collection. Finally, these findings cannot be generalized to other populations, as the sample originated from a single medical center.

## Conclusions

There is a high prevalence of meniscal tears and articular cartilage injuries in patients undergoing primary and revision ACLR. Up to 69.83% of the patients presented with meniscal tears, although the tear type differed between primary reconstruction and revision cases.

The overall prevalence of articular cartilage injuries was 24.48%, and the likelihood of sustaining these injuries was influenced by sex, age, the presence of meniscal tears, and whether the surgery was a revision procedure. Moreover, the present study delineates the severity and anatomical distribution of these lesions. Special attention should be paid to the medial femoral condyle, as it was the most frequently affected site, while the lateral tibial plateau was the least frequently involved.
